# Preoperative disinfection of foot and ankle: microbiological evaluation of two disinfection methods

**DOI:** 10.1007/s00402-018-2996-8

**Published:** 2018-07-10

**Authors:** Siem A. Dingemans, Ingrid J. B. Spijkerman, Merel F. N. Birnie, J. Carel Goslings, Tim Schepers

**Affiliations:** 1 Trauma Unit, Department of Surgery, Academic Medical Center, Meibergdreef 9, PO Box 22660, 1100 DD Amsterdam, The Netherlands; 2 Department of Clinical Microbiology, Academic Medical Center, Amsterdam, The Netherlands

**Keywords:** Disinfection, Alcohol, Chlorhexidine, Microbiology, Foot and ankle surgery

## Abstract

**Background:**

The aim of the current study was to investigate the effect of a footbath in alcohol prior to preoperative disinfection on bacterial flora of the foot and ankle.

**Methods:**

Twenty-two volunteers underwent skin preparation mimicking pre-surgical disinfection. One foot was submerged in a bag filled with 70% ethanol containing 10% IPA for 5 min after which it was painted with regular 0.5% chlorhexidine in 70% alcohol. The other foot was only painted with 0.5% chlorhexidine in 70% alcohol. Swabs were taken at four locations: (1) under the nailfold of the first toe, (2) first webspace, (3) sinus tarsi and (4) pre-tibial. A quantitative and qualitative analysis of the cultures was performed.

**Results:**

No statistically significant difference between the number of positive cultures between the two methods was observed. The number of colony forming units was statistically significantly lower on two locations in the footbath group (i.e., subungual and the first webspace) (median 1 versus median 92 *p* =0.03 and median 0 versus median 1 *p* =0.03, respectively). The number of cultures with heavy growth was lower in the footbath group under the nailfold of the first toe (5 versus 13 *p* =0.008). Thirty-eight different microorganisms were cultured.

**Conclusion:**

A footbath in alcohol prior to regular preoperative skin antisepsis significantly reduces the amount of bacteria under the nailfold and in the first webspace. The number of cultures with heavy growth is lower after a footbath in alcohol.

**Level of evidence:**

IV.

## Introduction

Surgical site infections (SSI’s) are a major concern as they account for a significant amount of morbidity, mortality and prolonged hospitalization [1–4]. Since the discovery of microorganisms, various methods of preventing microorganisms from colonizing surgical wounds have been developed. These methods vary from surgical dressings and sterilized tools to special air management in the operating room. As the human skin contains up to one trillion commensal bacteria, it is an obvious source of SSI’s [5]. It is conceivable that elimination of microorganisms from the skin will result in a reduction of SSI.

Skin antisepsis is even more important in trauma/orthopedic procedures of the foot and ankle as in this type of surgery the rate of SSI’s is higher compared to other parts of the body [6–9]. Examples are calcaneal fracture surgery with infection rates up to 33% worldwide and an average of 12.1% in Europe [10]. In ankle fractures which need open reduction and internal fixation, an infection rate of 4.3% is described [11]. In implant removal, especially on the lower extremity, SSI rates above 10% are reported [12]. The foot and ankle differ in many ways from other parts of the body by having less apocrine, but more eccrine glands, an alkaline environment, being heavily ridged, containing less pilosebaceous units (structure consisting of hair, hair follicle, arrector pili muscles, sebaceous gland) and being covered by socks and shoes most of the time creating a warm and moist environment which is ideal for growth of microorganisms [7, 13].

The most frequently used substances for preoperative skin cleansing are: (1) alcohols, (2) chlorhexidine (CHG), (3) povidone–iodine (PVI) or a combination of these [14]. All these substances have different qualities, some of which make them more suitable while others make them less favorable. Alcohol is microbiologically the most active; it is ten times more potent than CHG and PVI when it comes in its therapeutic concentration between 70 and 90% [14]. Chlorhexidine also has a direct effect, but has, in contrast to alcohol, a longer residual effect for up to 6 h. Furthermore it is active over a wide pH (5–8) when used within the correct therapeutic concentrations (0.5–3.5%) [7, 14–16]. PVI usually comes in an aqueous solution with its therapeutic concentration between 5 and 10% or between 0.5 and 1% ‘available’ iodine. It is active within a narrow pH (≈ 6) and needs to dry first before it becomes active [17]. Traditionally the administration of these substances is performed by painting of the surgical field. However, Ng et al. have shown a reduction in bacterial isolation after a preoperative chlorhexidine footbath [18]. Since alcohol is microbiologically active, we hypothesized an alcohol footbath could lead to an even larger reduction. To the best of our knowledge, alcohol has never been investigated as a footbath. Therefore, we combined the footbath with the common practice.

Keeping the unique properties of each substance in mind, we hypothesized that a combination of alcohol footbath with chlorhexidine would provide better preoperative skin antisepsis compared to traditional preoperative skin antisepsis. Our primary research question was: does a preoperative regime of bathing the foot and ankle in alcohol for 5 min have an additive value in bacterial load reduction. Secondary research question was: which organisms remain after preoperative skin antisepsis.

## Methods

Healthy volunteers took part in this pilot study. All volunteers provided full informed consent for taking part in this study. After obtaining informed consent, they underwent skin preparation mimicking pre-surgical skin decontamination. Excluded were volunteers with onchomycosis, paronychia, nail abnormalities, immune deficiencies or recent antibiotic use.

### Disinfection

Volunteers were treated as patients and disinfected on the operation table under the plenum. At first the right foot was submerged in a 1 l solution of 70% ethanol containing 10% isopropyl alcohol (IPA) (Orphi farma, Dordrecht, The Netherlands) in a sterile bag (Vi-Drape, MCD, MN, USA) for 5 min (Photo 1). It was made sure that both malleoli were just submerged.
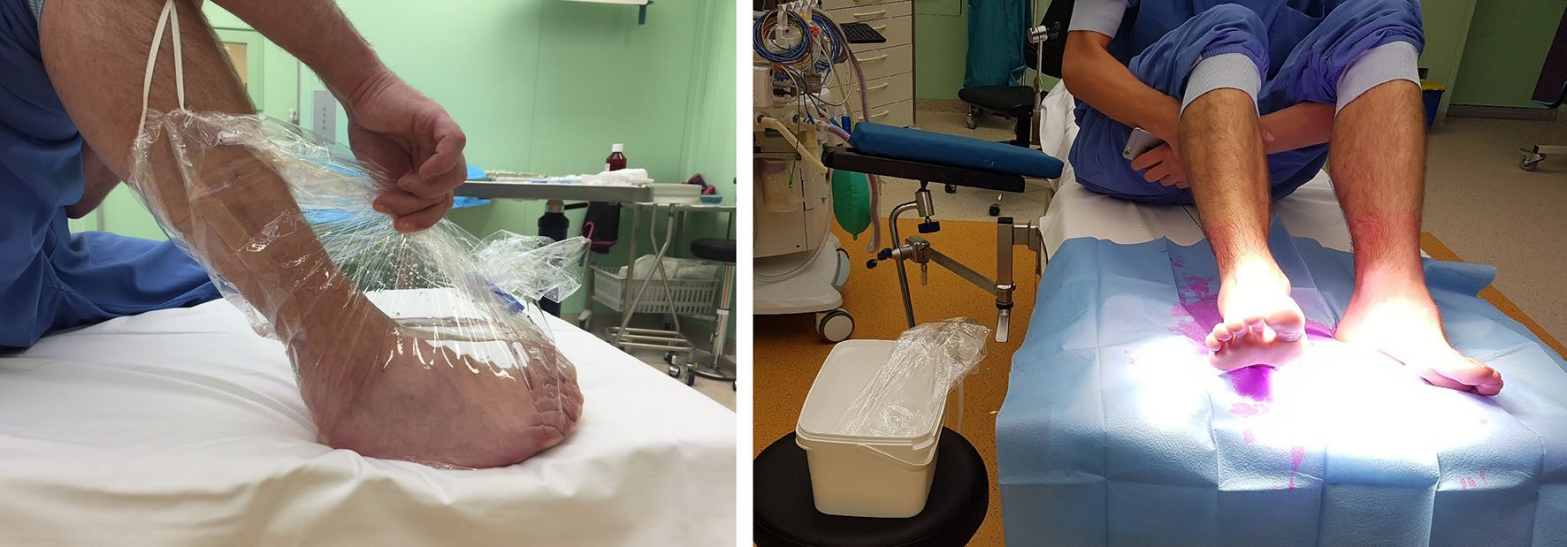


After this the left foot, ankle and half of the lower leg were painted with chlorhexidine 0.5% in 70% alcohol (Denteck, Zoetermeer, The Netherlands) for 2 ½ min using a sterile cotton on a sterile surgical clamp. In total, three cottons were used for the whole foot. The left foot was subsequently placed on a sterile field and left to dry. After 2 ½ min, the foot had dried and swabs were taken.

When the 5 min had passed, the right foot was placed on the sterile field as well and was left to dry. After it had dried, the lower right leg, ankle and foot were painted with chlorhexidine 0.5% in 70% alcohol for 2 ½ min using a new sterile surgical clamp and three sterile cottons as well. After 2 ½ min, the foot had dried and swabs were taken.

### Culture swabs

The culture swabs were taken after 2 ½ min after the feet were painted with chlorhexidine. From each lower extremity, culture swabs were taken from four locations using e-swabs (Copan, Brescia, Italy). The four locations were: (1) under the nailfold of the first toe, (2) the first webspace, (3) over the sinus tarsi and (4) pre-tibial. These were chosen because they are frequently operated on. Location 4 was chosen to serve as a control site because it was not covered by the alcohol, but painted on both sides. Each location was swabbed for 5 s.

### Cultures

Specimens were sent to the laboratory for quantitative aerobic and anaerobic culture. The e-swabs were pressed to the wall of the container and removed. The remaining fluid was mixed and 100 µl was inoculated on each agar plate. Several agar plates (Biomerieux, Marcy l’Etoile, France) were inoculated for aerobic and anaerobic culture for 2 and 6 days, respectively. All microorganisms were identified by mass spectrometry (Malditof; Bruker, Karlsruhe, Germany) and counted. The incubation period for the cultures was at least 7 days.

### Outcome

Cultures were analyzed both in a quantitative matter as a qualitative matter. Quantitative analysis was performed by determining the number positive cultures and number of colony forming units (CFU) of each culture. Furthermore, a distinction was made between cultures with light (i.e., < 20 CFU’s) and heavy (i.e., ≥ 20 CFU’s) growth [19]. Qualitative analysis was performed by determination of microorganisms cultured.

### Statistical analysis

Categorical variables are presented as counts and percentages, continuous variables are presented as means and standard deviations or medians and interquartile ranges (IQR) where appropriate. Normality was assessed using histograms and plots. McNemar’s tests for related samples were used to compare categorical data, *t* test for related samples or Wilcoxon signed rank test was used for continuous data where appropriate. All calculations were performed using SPSS v 23.0 (IBM, Chicago, Ill).

## Results

In total 22 volunteers took part in the study. All the cultures of the first volunteer were negative, which appeared to be a result of insufficiently dried paint. The cultures of this volunteer were, therefore, excluded from the study. For two volunteers, one culture was missing due to erroneous collection of the culture. Of the 21 volunteers included in the study, there were 13 (62%) females and eight (38%) males. After completion of the footbath and subsequent drying, there was no skin reaction, like signs of softened skin or wrinkling.

In total there were 87 positive cultures (out of 166). For the three test sites to be compared (site 1, site 2 and site 3), there were 75 positive cultures (out of 126). In the intervention (footbath) group there were 30 positive cultures, in the control group there were 35; this difference was not statistically significant (*p* = 0.90) (Table 1). The results for the quantitative analysis of the cultures are displayed in Table 2. Notably, the amount of CFU’s was statistically significantly lower in the footbath foot on location 1 (subungual) and location 2 (first webspace). The difference in number of cultures with heavy growth (> 20 CFU’s per site) is depicted in Table 3. The number of cultures with heavy growth was statistically significantly lower in the footbath foot on location 1 (subungual).

**Table 1:** Number of positive cultures per site

	Intervention (*n* = 21)	Control (*n* = 21)	*p* value
Subungual (%)	15 (75)	19 (91)	0.45^a^
First webspace (%)	10 (48)	14 (67)	0.29^a^
Sinus tarsi (%)	5 (24)	2 (10)	0.45^a^
Pre-tibial (%)	12 (57)	5 (24)	0.02^a^

^a^McNemar’s test for related samples

**Table 2:** Number of CFUs per site

	Intervention (*n* = 21)	Control (*n* = 21)	*p* value
Subungual (median) [IQR, range]	1 [0–1, 0–440]	92 [8–360, 0–1370]	0.03^a^
First webspace (median) [IQR, range]	0 [0–0, 0–7]	1 [0–13, 0–174]	0.03^a^
Sinus tarsi (median) [IQR, range]	0 [0–1, 2, 0–2]	0 [0–0, 0–1]	0.36^a^
Pre-tibial (median) [IQR, range]	0 [0–3, 0–81]	0 [0–0, 0–535]	0.26^a^

*IQR* interquartile range

^a^Wilcoxon signed rank test for related data

**Table 3:** Number of cultures with heavy growth (> 20 CFU’s per site)

	Intervention (*n* = 21)	Control (*n* = 21)	*p* value
Subungual (%)	5 (24)	13 (62)	0.008^a^
First webspace (%)	0 (0)	2 (10)	0.50^a^
Sinus tarsi (%)	0 (0)	0 (0)	0.99^a^
Pre-tibial (%)	1 (5)	1 (5)	0.99^a^

*CFU* colony forming unit

^a^McNemar’s test for related samples

In total, 38 different microorganisms were cultured. There were aerobic Gram-positive bacteria, aerobic Gram negative bacteria, anaerobic bacteria and fungi. Results from the qualitative analysis are displayed in Table 4.

**Table 4:** Qualitative analysis of cultures

Type	Subtypes
Aerobic Gram-positive bacteria	Coagulase-negative staphylococci
	Streptococci
	Micrococcalis
	Bacillus
	Corynebacteria
Aerobic Gram negative bacteria	Moraxella
	Haemophilus
	Nonfermenters
Anaerobic bacteria	Propionibacterium
	Clostridium
	Actinomyces
Fungi	Fungi (not determined)

## Discussion

With our study, we have shown that a 5 min footbath in alcohol prior to conventional preoperative disinfection of the foot/ankle reduces the bacterial inocula significantly under the nailfold of the first toe and the first webspace. Moreover the number of cultures with heavy growth is lowered under the nailfold. The overall number of positive cultures is not lowered by the preceding footbath. We took swabs only at an early stage to investigate whether or not a reduction in bacterial load was visible due to the difference in the preparation of skin antisepsis. Performing swabs during the procedure would provide information on the quality of maintaining a sterile field and not about the quality of the preoperative disinfection. Most likely re-taking cultures at a later stage would show an increase in bacterial load, which would probably be the same in both feet. Numerous studies have been conducted to identify the optimal method for preoperative skin antisepsis since skin antisepsis became routine in the late 1990s [20]. Traditionally iodine was used, it is; however, less attractive due to its irritant nature to the skin and it seemed to be less effective compared to chlorhexidine [17, 21]. Some studies suggest, however, that the advantage of CHG over PVI in these studies could be explained by the fact that PVI was an aqueous solution and CHG was diluted in alcohols [9, 14, 22]. They suggest that the actual active ingredient was alcohol and most beneficial effects could, therefore, be attributed to the alcohol [14]. The foot differs from others parts of the body and consists of several areas (i.e., under the nailfold and the webspaces) which are very difficult to disinfect as shown by previous studies [8, 19, 23–25]. Additional measures that are often used to further lower the chance of contamination the surgical site are, for example, covering the toes shows. However, only one small (underpowered) RCT is available on this subject; in this trial no benefit of covering the toes was observed [26]. Inspired by the study of Keblish et al. [19] who found good results in foot/ankle skin preparation using a bristled brush and solely alcohol and by the study of Becerro et al. [8] and Ng et al. [18] who made use of a footbath, we decided to use the best of both worlds. As alcohol evaporates quickly, we used a closed system footbath to overcome this problem. Despite using two substances, we still have observed high rates of positive cultures following skin antisepsis. These figures are, however, confirmed by earlier studies indicating that decontamination of especially the foot is challenging [8, 17, 23]. Skin antisepsis is even more important in light of increasing antibiotic resistance of microorganisms. If wound infections can be prevented, less antibiotics are needed and hence, less antibiotic resistance will develop. Different parts of the body naturally host different species of commensal bacteria [27]. The microorganisms most frequently cultivated from the foot/ankle by several investigators are Staphylococci [13, 17]. This is important as Gram-positive bacteria are a common source of SSI’s [7]. Interestingly we did not culture any *S. aureus* indicating that adequate skin antisepsis may prevent postoperative wound infections by eliminating important pathogens. Coagulase-negative Staphylococci (CoNS) are also frequently encountered in postoperative wound infections following osteosynthesis [17] and we did culture this microorganism often (in 30 out of 87 positive cultures); this finding has been observed by others earlier as well [8, 23]. Interestingly CoNS was almost exclusively cultured under the nailfold and the first webspace in both the intervention as the control group, Indicating that potential pathogens are hard to eliminate from these spots. Again, this has also been noted by other authors [8, 19, 23].

We contribute our finding that more positive cultures were found on site 4 in the experimental group (control site only disinfected with chlorhexidine paint) to the fact that this site was not covered by the alcohol and may have been less thoroughly disinfected in the testing environment. Furthermore, it must be noted that 4 of the 12 positive cultures on this site contained only one CFU which may indicate a doubtful clinical significance of this finding.

The main weakness of this study is that the direct relation between bacterial inocula and postoperative wound infections is yet to be proven. It is conceivable that reducing the bacterial load is beneficial for preventing wound complications; however, this is to be proven in clinical studies [15, 16, 26]. A pilot study in patients undergoing foot/ankle surgery should be the next step. With an infection rate of 10% and a reduction of 25%, a total of 6424 patients are needed. For a reduction of 50%, 864 patients would be necessary, both with an alpha of 5% and a power of 80%. Furthermore, the number of surgical site infections should serve as the primary endpoint.

The results of our study confirm our hypothesis that a footbath with alcohol is of additive value in foot/ankle skin preparation. We feel that adding this extra layer of decontamination could be valuable in the future because of the total amount of microorganisms was lower in the areas (i.e., nailfold and webspace) which are notoriously difficult to decontaminate and the number of cultures with heavy growth was also lower on these two locations. The effect in lowering SSI’s, however, needs to be proven in a clinical trial. As several authors suggested that alcohol might actually be the most active agent, the next study could be to investigate the effects of applying solely a footbath of alcohol compared to traditional chlorhexidine in alcohol paint.

## Conclusion

A footbath in alcohol prior to regular preoperative skin antisepsis significantly reduces the amount of bacteria under the nailfold and in the first webspace. Furthermore, the number of cultures with heavy growth is lower. Additional research is needed to differentiate the effect of the alcohol from the effect of the chlorhexidine and to investigate whether there is a clinical benefit in lowering surgical side infections.
